# Post-disappearance scenarios: policy implications following the potential disappearance of B/Yamagata lineage influenza viruses

**DOI:** 10.2807/1560-7917.ES.2024.29.45.2400196

**Published:** 2024-11-07

**Authors:** Marco Del Riccio, Marta C Nunes, Benjamin J Cowling, Bruno Lina, John W McCauley, Adam Meijer, Hanna Nohynek, Bronke Boudewijns, Saverio Caini

**Affiliations:** 1 Department of Health Sciences, University of Florence, Florence, Italy; 2 Netherlands Institute for Health Services Research (Nivel), Utrecht, the Netherlands; 3 Center of Excellence in Respiratory Pathogens (CERP), Hospices Civils de Lyon, and Centre International de Recherche en Infectiologie (CIRI), Université Claude Bernard Lyon 1, Inserm U1111, CNRS UMR5308, ENS de Lyon, Lyon, France; 4 South African Medical Research Council, Vaccines & Infectious Diseases Analytics (VIDA) Research Unit, Faculty of Health Sciences, University of the Witwatersrand, Johannesburg, South Africa; 5 World Health Organization Collaborating Centre for Infectious Disease Epidemiology and Control, School of Public Health, The University of Hong Kong, Hong Kong Special Administrative Region, China; 6 Virpath Lab, Centre International de Recherche en Infectiologie (CIRI), Université Claude Bernard Lyon 1, Inserm U1111, CNRS UMR5308, ENS de Lyon, Lyon, France; 7 Worldwide Influenza Centre (WIC), The Francis Crick Institute, London, United Kingdom; 8 Centre for Infectious Disease Control, National Institute for Public Health and the Environment (RIVM), Bilthoven, The Netherlands; 9 Finnish Institute for Health and Welfare (THL), Helsinki, Finland

**Keywords:** Influenza B/Yamagata, surveillance, public health, laboratory safety, vaccines, TIV

## Abstract

The COVID-19 pandemic and related preventive measures reduced influenza virus circulation, notably causing the disappearance of the B/Yamagata lineage of influenza viruses. In this Perspective, we discuss the implications that this development may have for global influenza epidemiology, and the adjustments that may need to be implemented concerning surveillance strategies and practices, laboratory safety protocols, and influenza vaccine formulations. The disappearance of the B/Yamagata lineage might indeed alter the dynamics of the influenza disease burden (although in a way that is difficult to predict at the moment), and associated diagnostic practices, and may also necessitate updated biosafety levels and revised influenza surveillance strategies. Furthermore, the World Health Organization (WHO) recommended in September 2023 the exclusion of B/Yamagata antigens from future vaccines, with a shift towards trivalent vaccines or modified quadrivalent vaccines; this new scenario underscores the importance of robust global respiratory virus surveillance, effective communication with healthcare professionals and the population to maintain trust in vaccines, and a collaborative approach among health policymakers and vaccine manufacturers to navigate this epidemiological change.

## Background

The emergence of the severe acute respiratory syndrome coronavirus-2 (SARS-CoV-2) and the subsequent widespread implementation of non-pharmaceutical interventions aimed at reducing its transmission and containing the spread of the epidemic resulted in a substantial decrease in the circulation of influenza and other respiratory viruses. Early after the relaxation of COVID-19 control measures, it was noted that influenza viruses of the B/Yamagata lineage had not resumed circulation [1]. This observation was supported by surveillance data, which consistently showed an almost completely absent circulation, suggesting that the B/Yamagata virus may have become extinct in the wild population [2]. The lower reproductive number of B/Yamagata influenza virus may have pushed it towards disappearance, while B/Victoria has continued to circulate, possibly thanks to the better fitness of its haemagglutinin deletion variants [3,4]. A comprehensive examination of the potential implications of the end of its circulation remains inconclusive at this stage, and while there is ongoing discussion, definitive guidance has yet to be determined. We believe addressing this issue is urgent; therefore, we discuss here the possible implications—such as changes in surveillance strategies, necessary revisions to laboratory practices for handling B/Yamagata, and vaccination strategies —and propose appropriate actions.

## Impact on global influenza burden

Forecasting the full impact of the potential disappearance of the B/Yamagata lineage on global circulation patterns and disease burden of influenza represents a complex challenge. Before 2020, the distribution of influenza B lineages exhibited distinct demographic and geographical patterns. The B/Victoria lineage was common across all age groups but tended to be more prevalent among younger populations (to children and adolescents) and in tropical regions, while the B/Yamagata lineage was widely distributed across age groups, including people older than 65 years, and was more frequently identified in temperate climates [5,6]. While no significant age-adjusted differences in terms of clinical presentation or disease severity between infections by the two influenza B lineages have been noted [7], the unique aforementioned epidemiological and virological characteristics of B/Yamagata must be considered when estimating the impact of its disappearance on global influenza burden. In fact, the B/Yamagata lineage exhibited slower antigenic drift and less frequent amino acid changes in its haemagglutinin protein compared with the B/Victoria lineage, resulting in reduced immune escape and a more stable immune response in the past years [8]. It is uncertain whether the absence of the B/Yamagata lineage will result in a diminished burden, its substitution by another pathogen akin to the replacement of influenza A(H1N1) by A(H1N1)pdm09 in 2009 [9], or if such displacement has already occurred due to the emergence of SARS-CoV-2; no doubt, the interplay with SARS-CoV-2 has introduced an additional layer of complexity and further curbed our ability to predict future epidemiological scenarios.

## Impact on laboratory practices

If the disappearance of the B/Yamagata lineage is confirmed, it would affect all laboratories that handle specimens containing the B/Yamagata component. Should B/Yamagata no longer be considered a contemporary human influenza virus, it would necessitate a thorough re-evaluation of the biosafety levels (BSLs) and imply handling the virus with increased precaution due to the progressive erosion of population immunity, mirroring practices for non-circulating influenza viruses such as A(H2N2) [10]. In particular, the adoption of more stringent containment measures, including enhanced BSL-2 practices, could be considered for handling B/Yamagata-lineage influenza viruses. Moreover, if the disappearance of B/Yamagata viruses in the population is confirmed, a transition to BSL-3 may become necessary to ensure increased safety and to align with the recommended precautions for non-circulating viral strains [11]. From a diagnostic standpoint, when B/Yamagata viruses have very low or no circulation, obtaining false-positive results becomes a major issue for accurate virus identification, due to a lower positive predictive value. This necessitates a re-evaluation of testing protocols to ensure high accuracy and reliability in influenza diagnostics and surveillance and highlights the need for safe synthetic positive controls in surveillance laboratories.

## Challenges for declaring local or global end of circulation

There are other reasons, in addition to issues in diagnostics, why particular caution is required before B/Yamagata influenza virus can be declared as extinct in the wild. Eradication defined as the “*permanent reduction to zero worldwide incidence of infection caused by a specific agent*” [12] could be used as an example for what is needed to declare B/Yamagata influenza virus extinct in the wild. To date, only the variola virus (causing smallpox) and the rinderpest virus have been globally eradicated. For other infectious agents, efforts either led to their elimination (reducing incidence to zero in specific areas, with ongoing measures to prevent re-establishment) in some countries or larger geographical areas but not globally, or proved unsuccessful in achieving even this less ambitious goal. 

Since influenza viruses are constantly evolving, criteria for declaring local or global end of circulation of B/Yamagata were never specified, and it is unclear what time period or criteria are required before this can be declared. Influenza B viruses possess the one key requirement for making extinction theoretically possible [13], which is the absence of significant non-human reservoirs or vectors. Yet, influenza B viruses circulate globally and evolve constantly via antigenic drift; the illness they produce is clinically indistinguishable from infections caused by other respiratory viruses, and vaccines are currently far from optimally effective in preventing infection and transmission. The combination of these characteristics meant that the extinction of type B influenza viruses was not considered a realistic objective before the COVID-19 pandemic [14]. While concerted and systematic efforts were and are made for the purpose of eradicating the viruses causing smallpox and poliomyelitis, the end of circulation of B/Yamagata viruses would represent the unforeseen and unintended consequence of public health measures that were devised and implemented with a different aim in view. For the first time, there might be a possibility to define criteria for declaring an influenza virus as extinct in the wild. This outcome was possibly favoured by lower circulation before 2020, in turn a consequence of the 2017/18 global B/Yamagata epidemic, the emergence of new influenza B viruses of the B/Victoria lineage, and the introduction of quadrivalent vaccines a few years before the COVID-19 pandemic. 

Finally, the seemingly overwhelming support provided by current epidemiological data to the hypothesis of B/Yamagata influenza virus disappearance must be counterbalanced by the consideration that the virus could still be circulating although at an extremely low level and therefore merely go undetected, while not yet gone extinct in the population. This could be the case if virus circulation were restricted to limited geographical regions in which surveillance is low or absent, especially considering the still low proportion of influenza B viruses for which the lineage is determined [15]. In addition, it is not implausible that influenza B/Yamagata could re-emerge at some point in the future, just as influenza A(H1N1) re-emerged in 1978 two decades after it had been displaced by A(H2N2) [16,17].

## Impact on vaccination

The most obvious consequence of the disappearance of B/Yamagata influenza viruses inevitably concerns influenza vaccines. In the recommendations for the composition of vaccine for the season 2024 (southern hemisphere) and 2024/25 (northern hemisphere), the World Health Organization (WHO) influenza advisory committee concluded that the inclusion of a B/Yamagata antigen in the vaccine is no longer warranted [18,19]. In fact, the committee went so far as to state that “*every effort should be made to exclude it as soon as possible*” from the vaccines [17], to contain the theoretical risk of reintroduction into the population of a virus no longer circulating in the wild, also considering that most, if not all, virologically confirmed B/Yamagata detections between 2020 and 2023 were due to the attenuated viruses in the live-attenuated influenza vaccines. The cross-protection conferred by the vaccine B/Victoria viral strain, although moderate, is a further element supporting this decision [20,21]. 

While we agree with the WHO recommendation, we hold that a decision of this type has profound implications, and any changes need to be carefully considered, managed and communicated. Whatever decisions are made, a concerted effort of public health authorities, policymakers, regulators, and vaccine manufacturers is essential to ensure that the risk of vaccine supply shortages due to the reversion of production processes from quadrivalent to trivalent vaccines is averted. Lastly, effective communication from governments, health authorities, and healthcare professionals in charge of administering the vaccine (e.g. general practitioners) is necessary to pass on the concept that any return to trivalent vaccines or changed formulation of quadrivalent vaccines does not negate the value of past formulations but is a progress reflecting an evidence-based response to the changing epidemiological landscape. Ensuring that the public understands this key concept will hopefully help avoid undermining the trust of the public, prevent misconceptions about the effectiveness of influenza vaccines, and avert any declines in vaccine uptake.

## Conclusion

We are faced with the possible disappearance of an influenza B virus lineage, an event that is not historically new and requires the timely adoption of strategies at different levels. Once more, a key recommendation is for the global network for respiratory virus surveillance to be strengthened and expanded, with particular emphasis on countries where surveillance systems have yet to achieve optimal functionality. Given the lessons learned from the COVID-19 pandemic, reinforcing the global respiratory virus surveillance network would serve preparedness and alert purposes whose importance far exceeds that of providing the evidence necessary to declare the end of circulation of the B/Yamagata lineage influenza virus. Continuous virological monitoring will be essential also to determine whether and how to modify laboratory procedures for handling B/Yamagata-lineage influenza viruses. Finally, while the WHO has taken action with regard to influenza vaccines, this is so far limited to recommending the exclusion of B/Yamagata antigens in inactivated vaccines and viruses in live-attenuated influenza vaccines from next-season vaccines. A debate aimed at devising a long-term, viable strategy for future influenza vaccines must be launched without delay, and it shall be open to all relevant stakeholders and include in-depth consideration of effective communication plans to support vaccination in the population.
